# Response to “Association between metformin use prior to admission and lower mortality in septic adult patients with diabetes mellitus: beware of potential confounders”

**DOI:** 10.1186/s13054-020-02918-2

**Published:** 2020-05-06

**Authors:** Huoyan Liang, Xianfei Ding, Tongwen Sun

**Affiliations:** General ICU, Henan Key Laboratory of Critical Care Medicine, Zhengzhou Key Laboratory of Sepsis, The First Affiliated Hospital of Zhengzhou University, Zhengzhou, 450052 China

To the Editor:

We thank Dr. Honore and his colleagues for their attention on our study in Critical Care [1]. First, we agree with their point that RRT has a protective effect on the mortality of patients with metformin (MET)-associated lactic acidosis [2]. However, there are some reasons to demonstrate that the lower mortality in septic patients with diabetes is due to MET treatment, rather than the metformin eliminated by RRT. First, the study of Doenyas-Barak et al. [3], one of the included studies in our meta-analysis [1], showed that the use of RRT between the MET-treated population and non-MET users was 38.6% and 21.2%, but there was no difference between the two groups (*p* = 0.13). More importantly, after removing this included study [3], we reworked the pooled effect of the remaining four studies and the result was consistent with our meta-analysis [1]. Furthermore, the study of Jochmans et al. [4] showed the use of RRT is higher in non-MET users than MET users (18.2% vs. 17.1%), but it also indicated that the protective effect of MET use in septic patients with diabetes. In addition, the metformin treatment can improve the liver injury and inflammatory response and even ameliorate the mortality of septic mice in our ongoing experimental study. Finally, we believe that studies in the future to assess the association between metformin and mortality in septic patients with diabetes will be performed.

## Data Availability

Not applicable.
